# Comparison of Chest X-rays findings in COVID-19 suspected and confirmed cases at a university teaching hospital: A retrospective comparative study

**DOI:** 10.12669/pjms.38.1.4624

**Published:** 2022

**Authors:** Misbah Durrani, Afshan Shahid, Ume Kalsoom, Inam-ul-Haq, Anum Yousaf, Saqib Naveed

**Affiliations:** 1 Dr. Misbah Durrani, MCPS. FCPS. MHPE; 2 Dr. Afshan Shahid, MPH, MSc, FCPS. Associate Professor Community Medicine, SIMS Lahore, Pakistan; 3 Dr. Ume Kalsoom, FCPS. Assistant Professor, RMU; 4 Dr. Inam ul Haq, FCPS. MHPE. Associate Professor, Al-Shifa Trust Eye Hospital, Rawalpindi, Pakistan; 5 Dr. Anum Yousaf, MBBS Residents, Department of Diagnostic Radiology, Benazir Bhutto Hospital, Rawalpindi, Pakistan; 6 Dr. Saqib Naveed, MBBS Residents, Department of Diagnostic Radiology, Benazir Bhutto Hospital, Rawalpindi, Pakistan

**Keywords:** COVID-19 patients, Chest X-rays (CXR), Corona virus, Reverse transcriptase polymerase chain reaction RT-PCR

## Abstract

**Objectives:**

To compare Chest X-rays findings in COVID -19 suspected and confirmed patients on RT-PCR, presented at corona filtration center, Benazir Bhutto hospital Rawalpindi.

**Methods:**

In this study, Chest radiographs of 100 COVID-19 RT-PCR positive confirmed patients were compared with 100 RT-PCR negative suspected COVID-19 patients screened at corona filtration center, Benazir Bhutto Hospital Rawalpindi from November 2020 to December 2020. Data on demographics, presenting complaints, co-morbid, lesion characteristic, distribution and attenuation, lobar involvement, pleural effusion and lymphadenopathy were collected. Associations between imaging characteristics and COVID-19 pneumonia were analyzed with univariate and multivariate logistic regression modals.

**Results:**

Chest X-rays findings revealed bilateral lung consolidation with peripheral and diffuse distribution, involving middle and lower lobe to be statistically significant (p<0.05) between RT-PCR positives and negative patients. Peripheral distribution was associated with an 11.08-fold risk in COVID-19 positive patients than diffuse distribution. Middle lobe involvement had four folds risk and lower lobe involvement had 11.04 folds risk in COVID-19 cases as compared to upper lobe involvement. Consolidation had 2.6 folds risk in COVID-19 positive cases.

**Conclusions:**

Bilateral, peripheral distribution of middle and lower lobes ground glass haze or consolidation with no pleural effusion is significantly related to COVID-19 pneumonia. Overlapping imaging features of the infectious and non-infectious COVID mimickers can be further excluded by detailed clinical evaluation and further radiological workup.

## INTRODUCTION

War against COVID-19 is still raging on as it continues its devastations and pose a major unprecedented health crisis globally. Although in an era of pandemic, every patient with respiratory symptoms like cough, dyspnea, sore throat and fever is considered to be having COVID-19, yet there are other infectious and non-infectious pulmonary diseases which may mimic COVID-19 radiologically.

HRCT (high resolution computed tomography) is the radiological investigation of choice as it has high sensitivity of 98% as compared to RT-PCR which has a sensitivity of only 60-71%.^1^ RT-PCR has also additional limitations regarding faulty sampling techniques, Kit performance and awaited results. Chest X-rays, although having sensitivity of almost 67% are still used in our public hospitals and rural health centers as first line of investigation as compared to HRCT. Chest X-rays are readily available, affordable, less time consuming in terms rigorous CT decontamination measures and also in terms of expert radiological interpretation by general practitioners and physicians.^2^ It was therefore thought to take RT-PCR as gold standard and to compare CXR findings of COVID 19 RT-PCT positive patients with that of COVID 19 RT-PCR negative patients having similar respiratory symptoms.

## METHODS

This is a retrospective descriptive study was conducted from November 2020 to December 2020 after consent from ethical review board. About 200 patients with clinical suspicion of COVID 19 who visited corona filter clinic at Benazir Bhutto hospital Rawalpindi were included. Chest X-rays are stored in central computer server of Benazir Bhutto hospital.Chest radiographs of 100 COVID-19 RT-PCR positive confirmed patients were compared with 100 RT-PCR negative patients.

### Inclusion criteria:

1. Patients with symptoms suggesting of COVID 19
2. COVID-19 RT-PCR result.

### Exclusion criteria:

1. Normal chest X-ray
2. Non COVID X-ray features like pneumothorax, pulmonary edema, lung cavities/calcifications
3. Post admission CT chest features suggestive of COVID 19.

Quantitative variables like age are presented as mean with standard deviation. Qualitative variables like gender and co morbidities were presented as frequency and percentages. Symptoms, CXR findings were also presented as frequency and percentages. Group difference and statistical significance was seen by applying chi-square test on all categorical variables. Univariate regression analysis was applied to see the effect size for the relationship among all variables in two groups. Lastly multivariate regression analysis was performed to evaluate the associations between different characteristics and outcome. Model-1 was created by not adjusting any variable, final model adjusted for age, gender, all symptoms, and co morbidities sequentially. A two-tailed p-value of less than .05 was taken as statistically significant. All analysis was done by using SPSS version 23.

### Ethical Approval:

Ref. No. 52 /IREF\RMU\2020, Dated: 14-04-2020.

## RESULTS

Two hundred patients were analyzed retrospectively in a tertiary care treatment public sector hospital for COVID-19 in Rawalpindi. Patients were divided in two groups and labelled as COVID-19 positive or negative on the basis of RT-PCR. Mean age of patients in COVID-19 positive group was 45±15.5 with 76% males and 24% females. Mean age of patients in COVID-19 negative group was 37±18.1 with 57% males and 43% females. Among the different characteristics of patients investigated in history, presence of co-morbidities and imaging characteristics on X-rays, significant difference was found in gender, history of fever and sore throat, and diabetes. Chest X-rays findings revealed bilateral lung consolidation with peripheral and diffuse distribution, involving middle and lower lobe to be statistically significant (p<0.05) between RT-PCR positives and negative patients. The details of patient’s characteristics are shown in Tables-I & II. Significant association between gender, history of fever, sore throat, diabetes, disease distribution, lobes involvement, consolidation and COVID-19 pneumonia were observed on univariate analysis as shown in Table-III.

**Table I:** Base line characteristics of patients. (Figures are presented as whole numbers with percentages in brackets).

Characteristics		COVID-19 Negative	COVID-19 Positive	p-value
***Age (years)***				
Mean ± SD		37±18.1	45±15.5	
***Gender***				
Male		57(57%)	76(76%)	0.004
Female		43(43%)	24(24%)	
***Smoking***				
	No	80(80%)	83(83%)	0.585
	Yes	20(20%)	17(%)	
***Symptoms***				
Fever	No	37(37%)	19(19%)	0.005
	Yes	63(63%)	81(81%)	
Cough	No	35(35%)	38(38%)	0.659
	yes	65(65%)	62(62%)	
Shortness of breath	No	60(60%)	55(55%)	
	Yes	40(40%)	45(45%)	0.474
Sore throat	No	69(69%)	13(13%)	
	Yes	31(31%)	87(87%)	0.002
Loss of smell	No	98(98%)	99(99%)	0.561
	Yes	2 (2%)	1(1%)	
Loss of taste	No	97(97%)	96(96%)	
	Yes	03(3%)	04(4%)	.67
Diarrhea	No	81(81%)	96(96%)	
	Yes	19(19%)	4(4%)	0.001
Travel history	No	61(61%)	82(82%)	
	Yes	39(39%)	18(18%)	0.001
***Co-morbidities***				
Diabetes	No	85(85%)	06(06%)	0.038
	Yes	15(15%)	94(64%)	
Hypertension	No	83(83%)	38(38%)	0.093
	Yes	17(17%)	62(62%)	
CKD	No	95(95%)	97(97%)	
	Yes	05(05%)	03(03%)	0.470
Tuberculosis	No	94(94%)	87(87%)	
	Yes	06(06%)	13(13%)	0.306
Asthma	No	100(100%)	97(97%)	0.081
	Yes	0 (0%)	03(03%)	
Cardiac problems	No	90(90%)	96(96%)	0.297
	Yes	10(10%)	04(4%)	
***Lung involvement***				
Unilateral (either right/ left lung involved)	No	89(89%)	87(87%)	
	Yes	11(11%)	13(13%)	0.663
Bilateral	No	69(69%)	23(23%)	
	Yes	31(31%)	77(77%)	0.00
***Disease distribution***				
Peripheral	No	64(64%)	33(33%)	
	Yes	36(36%)	67(67%)	0.00
Central	No	92(92%)	98(98%)	
	Yes	08(08%)	02(02%)	0.05
Diffuse	No	98(98%)	76(76%)	
	Yes	02(02%)	24(24%)	0.00
***Involvement of Lobes***				
Upper	No	96(96%)	96(96%)	
	Yes	04(04%)	04(04%)	0.639
Middle	No	80(80%)	56(56%)	
	Yes	20(20%)	44(44%)	0.00
Lower	No	66(66%)	13(13%)	
	Yes	34(34%)	87(87%)	0.00
Lymphadenopathy	No	97(97%)	97(97%)	
	Yes	03(03%)	03(03%)	0.663
Pleural effusion	No	90(90%)	92(92%)	
	Yes	10(10%)	08(08%)	0.084
Lung fibrosis	No	98(98%)	97(97%)	
	Yes	02(02%)	03(03%)	0.651
***Attenuation***				
Ground glass appearance	No	70(70%)	33(06%)	
	Yes	30(30%)	67(94%)	0.648
Consolidation	No	78(78%)	36(36%)	
	Yes	22(22%)	64(64%)	0.02

**Table II:** Univariate analysis results. (Figures are presented as whole numbers with percentages in brackets)

Variables	Statistics	OR (95% CI)
Age (mean, SD)	45±15.5	
***Gender***		
Female	76(76%)	1
Male	24(24%)	2.38(1.30,4.3) ***
***Smoking History***		
No	83(83%)	1
Yes	17(%)	.88 (.63,1.89)
***Fever***		
No	19(19%)	1
Yes	81(81%)	2.5 (1.31,3.6) ***
***Cough***		
No	38(38%)	1
Yes	62(62%)	1.06 (.80,1.41)
***Shortness of Breath***		
No	55(55%)	1
Yes	45(45%	1.22 (.70,2.15)
***Sore Throat***		
No	13(%)	1
Yes	87(87%)	1.88 (1.17,3.04) ***
***Loss of smell***		
No	99(99%)	1
Yes	1(1%)	.495(.04,5.54)
***Loss of taste***		
No	96(96%)	1
Yes	4(4%)	.67(.06,4.73)
***Travel History***		
No	82(82%)	1
Yes	18(18%)	1.81 (1.20,2.72) ***
***Diabetes***		
No	94(94%)	1
Yes	06(6%)	1.6(1.2,3.1) **
***Hypertension***		
No	38(38%)	1
Yes	62(62%)	1.5(.87,2.6)
***Kidney Disease***		
No	97(97%)	1
Yes	03(03%)	1.3(.54,2.6)
***Tuberculosis***		
No	87(87%)	1
Yes	03(03%)	1.5(.59,3.8)
***Asthma***		
No	97(97%)	1
Yes	03(03%)	.492(.427,8.34)
***Cardiac Problems***		
No	96(96%)	1
Yes	04(04%)	1.3(.71,2.6)
***Lung Involvement***		
Unilateral	13(13%)	1
Bilateral	77(77%)	7.4(2.7,8.6) ^***^
***Disease distribution***		
Diffuse	24(24%)	1
Peripheral	67(67%)	11.08(5.03,15.08) ***
***Involvement of lobes***		
Upper	4(4%)	1
Middle	44(44%)	4.23(1.73,5.8) **
Lower	87(87%)	11.5(5.06,15.8) ***
***Lymphadenopathy***		
No	97(97%)	1
Yes	03(03%)	.87(.67,2.2)
***Pleural effusion***		
No	92(92%)	1
Yes	08(08%)	1.1(.60,1.94)
***Lung Fibrosis***		
No	97(97%)	1
Yes	03(03%)	1.5(.248,3.79)
***Ground glass appearance***		
No	67(67%)	1
Yes	33(33%)	1.07(.79,1.45)
***Consolidation***		
No	36(36%)	1
Yes	64(64%)	2.6(1.09,3.2) **

**Table III:** Multivariate Analysis-Relationship between X-Ray findings and COVID-19 status of patients.

Variables	Statistics	OR (95% CI)
***Involvement of lobes***		
Upper	4(4%)	1
Middle	44(44%)	4.1(2.3,5.1) ***
lower	87(87%)	11.5(5.06,15.8) ***
***Disease Distribution***		
Diffuse	24(24%)	1
Peripheral	67(67%)	11.08(6.23,16.1) ***
***Consolidation***		
No	36(36%)	1
Yes	64(64%)	2.6(1.09,3.2) ***

Final model adjusted for age, gender, all symptoms, and co morbidities sequentially.

*** highly significant statistically, *CI,* Confidence interval, OR Odds Ratio.

Different variables were adjusted according to the Likelihood ratio test depending upon their contribution in the model, peripheral distribution was associated with a 11.08-fold risk in COVID -19 positive patients than diffuse distribution. Middle lobe involvement had four folds risk and lower lobe involvement had 11.04 folds risk in COVID 19 cases as compared to upper lobe involvement. Consolidation had 2.6 folds risk in COVID -19 positive cases. The results of multi variate analysis are shown in Table-III.

## DISCUSSION

COVID-19 is a multifaceted disease with variable presentations and unpredictable outcomes. So, in an era of current pandemic, every patient with clinical symptoms of fever, dry cough, sore throat is considered to be COVID-19 positive, until proven otherwise. This puts a huge constraint on already limited and stretched out healthcare system especially in a third world country. This also stresses the need to optimally use our diagnostic resources. Portable chest X-rays although having a sensitivity of 55% at ≤2 days to 79% at >11 days^3^,^4^ is still the primary investigation of choice in terms of feasibility and cost effectiveness with minimal risk of cross infection and exposure to the paramedical staff. It has its limitation regarding its inability to distinguish between COVID 19 and other COVID 19 mimickers purely on Chest X-ray findings.^5^ Although HRCT chest is the ideal radiological investigation of choice with sensitivity of 98%,^6^ it is reserved for RT-PCR negative but symptomatic patients, patient’s with COVID complications like pulmonary embolism and acute respiratory distress syndrome, and for follow up of post COVID pulmonary fibrosis. CT chest is its own challenges regarding decontamination measures, affordability, and availability in primary health care centers and in terms of expert opinion.^7^

**Fig.1:**
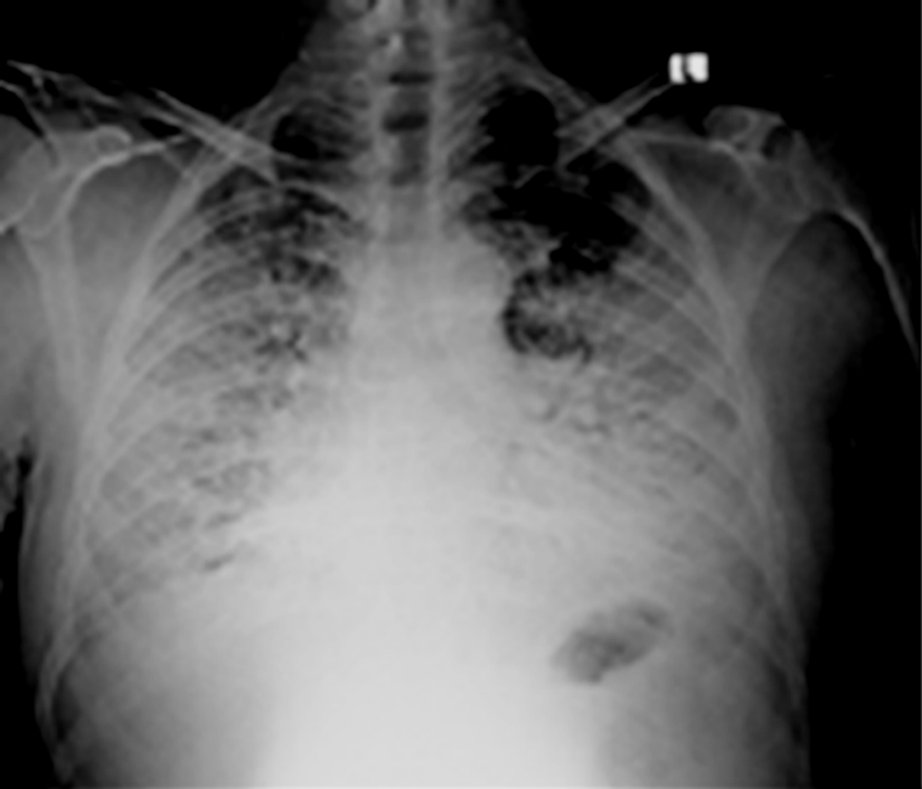
Chest X-ray of a COVID positive, middle aged female patient showing homogenous consolidation in bilateral lung fields predominantly in peripheral distribution in mid and lower zones with obscuration of cardiac and diaphragmatic silhouette and costophrenic angles on both sides. Patient had an acute episode of fever, shortness of breath and cough with no positive contact history.

Our study showed that bilateral, peripheral distribution of middle and lower lobes ground glass haze or consolidation with no pleural effusion is significantly related to COVID-19 pneumonia which is consistent with other studies^8^-^11^, however our study also showed that peripheral distribution was associated with an 11.08-fold risk in COVID-19 positive patients compared with diffuse distribution of other viral or bacterial pneumonias. Fig.1. Middle lobe involvement had four folds risk and lower lobe involvement had 11.04 folds risk in COVID 19 cases as compared to upper lobe involvement. Consolidation had 2.6 folds risk in COVID -19 positive cases. There are number of infectious diseases like atypical viral or fungal pneumonias that can mimic COVID 19 infection. There are also numbers of noninfectious diseases like pulmonary edema, hemorrhage, alveolar proteinosis, interstitial lung disease, organizing pneumonias or aspiration pneumonias that can be difficult to differentiate from COVID 19 infection on chest X-rays. There are studies^5^,^12^ to differentiate between these diseases on CT chest findings but literature on chest X-rays is lacking. In our study patients with Radiological findings were described according to Fleischner Society glossary of terms for Thoracic imaging.^13^ Ground glass opacities were defined as increased opacification of lung parenchyma not obscuring blood vessels and bronchi. Consolidation was described as homogenous opacification of lung parenchyma obscuring blood vessels and bronchi.

**Fig.2:**
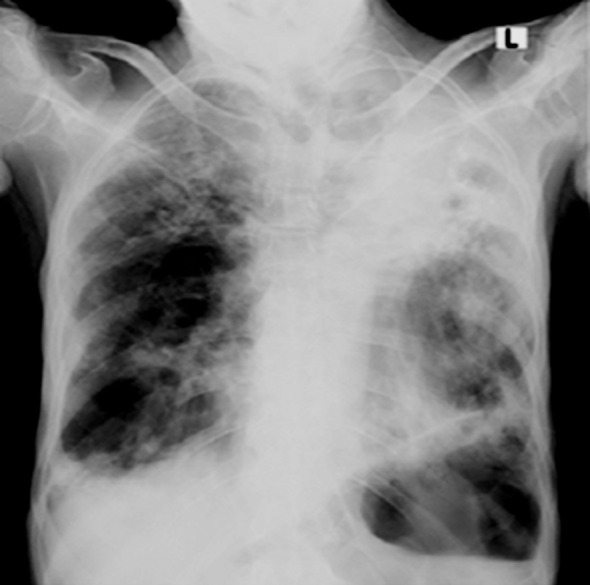
Chest X-ray of a young male patient, having cough, sore throat, rhinitis, shortness of breath and fever but had negative COVID serology. X-ray showed left upper lobe collapse, fibrocalcific changes, atelactatic bands in left lower zone, elevation of ipsilateral hemidiaphragm with few calcific granulomas in right middle zone, these are sequelae of healed pulmonary koch’s. (Consistent with History) In homogenous airspace shadowing in both upper and right mid zones, bilateral pleural effusions suggestive of acute overwhelming infection on background of chronic healed granulomatous disease. Patient was advised for gene expert test to rule out reactivation of pulmonary Koch’s.

Viral pneumonias show chest X- ray findings of unilateral or bilateral ground glass haze, patchy consolidations, bronchial wall thickening, reticulonodular infiltrates and small pleural effusions.^12^ COVID 19 pneumonia, severe acute respiratory syndrome virus (SARS) and middle east respiratory syndrome virus (MERS) all show subpleural distribution of consolidation or ground glass haze while there is generally absence of cavitation, pleural effusion and pneumothorax in COVID and SARS.^14^-^16^ Tuberculosis is the most common pulmonary infection in our subcontinent. Chest Xray finding of pulmonary tuberculosis are lobar or multilobar cosolidations, bronchopneumonias, pleural effusions, lymphadenopathy, miliary tubercles, cavitating lesions with bilateral upper lobe predilections. Ground glass haze is not a feature of pulmonary tuberculosis.^17^ Fig.2. Fungal pneumonias usually occur in immunocompromised patients. They may show cavitating lesion with fungus ball (air meniscus sign), dilated bronchi with gloved finger appearance or in cases of invasive aspergillosis areas of ground galls haze, cavitating consolidations, pleural effusions and lymph adenopathy. There is no zonal predilection.^18^

Interstitial lung disease tops the differential for COVID 19 infection. Patients with non-specific interstitial pneumonia and desquamative interstitial pneumonia may be indistinguishable from COVID-19 on chest X-ray findings alone as they also present with bilateral peripheral areas of ground glass haze with middle and lower zonal predominance. Patients of NSIP would have progressive history of dyspnea, CT chest findings of reticular opacities with subpleural sparing and volume loss. Patients with DIP would be chronic smokers with CT chest finding of bilateral reticular opacities and emphysematous changes. Hypersensitivity pneumonitis is another differential to be considered with Chest X-ray showing bilateral peripheral ground glass haze/consolidations with fine reticulations, however there are also poorly defined small nodular opacities sometimes sparing apices and bases.^19^

Patients with congestive cardiac failure and pulmonary edema show Chest Xray findings of cardiomegaly, upper lobe blood diversion, Kerly A and B lines, bat wing consolidation with air bronchograms, bilateral pleural effusions, interlobar thickening and peribronchial cuffing making it distinguishable from COVID 19 pneumonia. Patients with pulmonary hemorrhage will have history of collagen vascular diseases, trauma or anticoagulation therapy history. Chest X-ray finding would be of focal, multifocal areas of ground glass haze or consolidation with cavitations, pleural effusions with no zonal or lobar predilection.

Chest Xray findings of alveolar proteinosis are widespread nodular or reticulo nodular opacities or consolidation with relative apical and costophrenic angle sparing, but pleural effusions and lymph adenopathy is usually not a feature of this disease.

Organizing pneumonias show chest X–ray findings of bilateral subpleural., diffuse or patchy consolidations or reticulonodular opacities^20^, while drug induced organizing pneumonias may have varied findings such as wide spread consolidation, with interstitial fibrosis 21. Patients with aspiration pneumonia will have a pre-existing condition and show patch infiltrate in dependent lung segments with pleural effusions and cavitation.

### Limitations of the Study:

It is a study retrospective with small sample size. Chest X-ray severity index was not calculated and there was no co-relation between chest X-ray findings and CT chest findings.^3^, ^22^-^24^ There should be more comprehensive studies to differentiate between infectious and non-infectious COVID 19 radiological mimickers.

Another limitation was absence of serial CXRs to see progression of disease in terms of its resorptive phase and pulmonary fibrosis in our population.^25^ Clinical significance of this study to guide our general practitioners, clinicians, radiologists and paramedical health care workers especially working in primary health centers and clinics to diagnose COVID 19 on chest X-ray findings alone.^26^,^27^ The awareness and knowledge of diseases that mimic COVID 19 on chest X-rays can help in early management of these diseases and prevent unnecessary wastage of resources in times of pandemic.

## CONCLUSION

Bilateral, peripheral distribution of middle and lower lobes ground glass haze or consolidation with no pleural effusion is significantly related to COVID-19 pneumonia. Overlapping imaging features of the infectious and non-infectious COVID mimickers can be further ironed out/excluded by detailed clinical evaluation and further radiological workup.

### Authors’ Contribution:

**MD:** Conceived, designed, manuscript writing and is responsible for integrity of the study.

**UK:** Did data collection and manuscript writing.

**AS & IUH:** Designed methodology, performed statistical analysis and editing of manuscript.

**AY & SN** : Data collection.
